# All-lead-free Cs_2_SnCl_6_/Cu_2_ZnSnS_4_/CuFeO_2_ cascade band-aligned multilayer heterostructures for solar-driven hydrogen production from wastewater

**DOI:** 10.1039/d5na00828j

**Published:** 2025-11-24

**Authors:** Amira H. Ali, Ashour M. Ahmed, M. A. Basyooni-M. Kabatas, Mamduh J. Aljaafreh, Mohamed Shaban, Mohamed Rabia, Ahmed A. Abdel-Khaliek

**Affiliations:** a Nanomaterials Laboratory, Department of Chemistry, Faculty of Science, Beni-Suef University Beni-Suef Egypt; b Department of Physics, Faculty of Science, Imam Mohammad Ibn Saud Islamic University (IMSIU) Riyadh 11623 Saudi Arabia; c Department of Precision and Microsystems Engineering, Delft University of Technology Mekelweg 2 2628 CD Delft The Netherlands m.kabatas@tudelft.nl m.a.basyooni@gmail.com; d Department of Nanotechnology and Advanced Materials, Graduate School of Applied and Natural Science, Selçuk University Konya 42030 Turkey; e Department of Physics, Faculty of Science, Islamic University of Madinah Madinah Saudi Arabia; f Department of Chemistry, Faculty of Science, Beni-Suef University Beni-Suef Egypt

## Abstract

Lead-free halide perovskite, kesterite, and delafossite semiconductors were integrated into a multilayer ternary heterostructure (Cs_2_SnCl_6_/Cu_2_ZnSnS_4_/CuFeO_2_) to enable direct solar-driven hydrogen production from sewage water. X-ray photoelectron spectroscopy confirms the expected elemental composition and oxidation states, while X-ray diffraction verifies the successful incorporation of all three layers with well-defined crystallinity. Optical measurements reveal a systematic narrowing of the effective band gap, decreasing from 1.73 eV for CuFeO_2_ to 1.50 eV for the Cu_2_ZnSnS_4_/CuFeO_2_ bilayer and further to 1.12 eV for the complete Cs_2_SnCl_6_/Cu_2_ZnSnS_4_/CuFeO_2_ stack. The multilayered architecture enabled effective charge separation and transport, delivering a photocurrent density of −24.0 mA cm-2, approximately 77 times higher than the dark current density. The incident photon-to-current efficiency reaches 77%. These results demonstrate strong photoresponsivity and confirm the suitability of the multilayer heterojunction for efficient solar-driven hydrogen production. The extracted thermodynamic parameters (ΔH* = 3.452 kJ mol^−1^ and ΔS* = 9.644 J mol^−1^ K^−1^) indicate a low activation barrier for interfacial charge transfer, suggesting that the system effectively couples photonic and thermal contributions to enhance hydrogen-evolution kinetics. Collectively, these findings establish the all-lead-free Cs_2_SnCl_6_/Cu_2_ZnSnS_4_/CuFeO_2_ heterostructure as a highly efficient photoelectrode for solar-to-hydrogen conversion in complex wastewater environments. Demonstrating hydrogen evolution directly from sewage water further highlights the dual functionality of this architecture for simultaneous wastewater valorization and sustainable fuel production.

## Abstract 2

Lead-free CuFeO_2_/Cu_2_ZnSnS_4_/Cs_2_SnCl_6_ heterostructures enable solar-driven hydrogen production from wastewater, achieving 24.0 mA cm^−2^ photocurrent and 77% IPCE *via* efficient charge separation.

## Graphical abstract

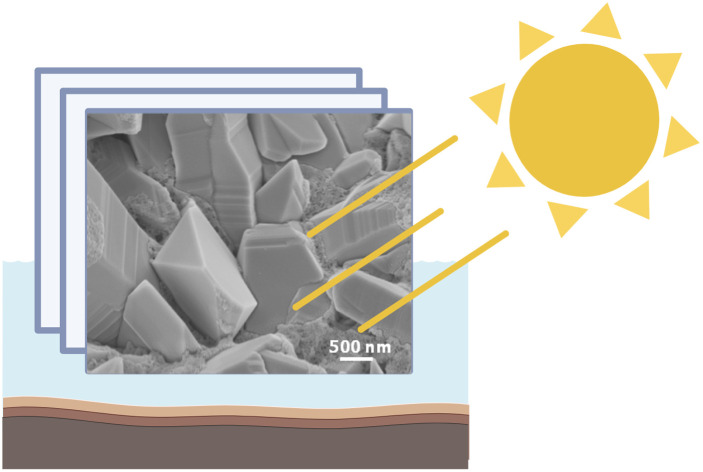

## 1. Introduction

Hydrogen gas is essential across diverse industrial sectors, including petroleum refining, glass purification, fertilizer production, metal processing, and advanced technologies for energy conversion and storage.^1–5^ More importantly, hydrogen is increasingly recognized as a cornerstone for the global transition toward renewable and sustainable energy systems. Among various technologies of hydrogen generation, PEC (photoelectrochemical) water splitting stands out as an eco-friendly route to drive the hydrogen evolution reaction (HER) using photocatalysts.^1–3^ In PEC systems, photocatalysts play a critical role in harvesting sunlight, generating photoinduced charge carriers, and facilitating redox reactions for HER. To perform effectively, photocatalysts must possess key features, including a suitable band gap for absorbing visible light, high chemical photostability, and a composition based on earth-abundant, non-toxic elements.^6–8^ Several classes of semiconducting materials have been explored for PEC applications, notably perovskites, delafossites, and kesterites, each offering distinct advantages.

Copper-based delafossite-type oxides (CuXO_2_, where X = Fe, Mn, Ga, Al, Cr, *etc.*) have attracted substantial attention due to their layered structure and strong covalent Cu–O bonds, which contribute to high hole mobility and chemical stability.^9–11^ For water splitting, CuFeO_2_ exhibits good optical absorption, an appropriate onset potential, and a low conduction band edge.^12–14^ Moreover, it benefits from ease of synthesis *via* low-temperature hydrothermal processes,^15,16^ making it an appealing and scalable choice for PEC applications.

Similarly, kesterite-structured semiconductors, such as Cu_2_ZnSnS_4_, have emerged as promising materials for photocatalytic and photovoltaic technologies. Cu_2_ZnSnS_4_ is made up of abundant elements on Earth and is non-toxic, making it an environmentally friendly and sustainable choice.^17,18^ It exhibits strong absorption in the visible spectrum. Moreover, the Cu_2_ZnSnS_4_ layer can be produced through various straightforward and economical techniques. Its photocatalytic performance can be further enhanced through surface modifications, enabling it to function as an efficient light harvester in PEC systems. Recent research has documented solar-to-hydrogen conversion efficiency reaching as high as 0.28% under AM 1.5G illumination using Cu_2_ZnSnS_4_-based photoelectrodes.^19^

Perovskites have also transformed the field of solar energy conversion, particularly in photovoltaics and optoelectronics.^20–22^ Lead-free metal halides (LFMHs), including double perovskites (A_2_B(iv)X_6_) and elpasolites (B(iii)X_6_), offer more sustainable efficiency in HER.^2,23–25^ However, elpasolites often suffer from wide band-gaps that limit solar absorption. Among the LFMHs, cesium tin chloride (Cs_2_SnCl_6_) has proven to be a promising material for PEC applications. Cs_2_SnCl_6_ exhibits exceptional optical characteristics, including minimal reflectivity, superior electrical conductivity, a substantial static refractive index, strong optical absorption, and a high dielectric constant.^26,27^ Its band gap is related to the transition at the lowest unoccupied conduction band, primarily driven by the movement of electrons from Cl-p orbitals to Sn-p orbitals. Density of states calculations suggest that Cs_2_SnCl_6_ acts as a p-type semiconductor.^26^ Moreover, this material exhibits notable stability in thermodynamic and thermal aspects. It can absorb a broad spectrum of light and supports the transport of charge carriers. Its elevated Seebeck coefficient and non-toxic properties make Cs_2_SnCl_6_ particularly promising for thermoelectric applications and spintronic devices.^26^

Building on the individual strengths of each component, photocatalysts integrating CuFeO_2_/Cu_2_ZnSnS_4_/Cs_2_SnCl_6_ multilayer offer a synergistic strategy to enhance PEC hydrogen production. These materials form well-aligned heterojunctions that facilitate efficient charge separation, suppress electron–hole recombination through internal electric fields, and optimize energy band alignment. Moreover, the multilayer structure enables extended light absorption across a broader range of the solar spectrum, thereby improving photon utilization. These synergistic interactions enhance photocatalytic activity, improve long-term operational stability, and cost-effectiveness.

Copper foil was selected as the substrate for multilayer deposition because of its high electrical conductivity, which supports rapid charge transport and efficient electron collection in PEC photoelectrodes.^28,29^ Its strong thermal conductivity and mechanical compatibility with diverse semiconductor films further enhance stability and performance. Combined with its low cost, these properties make copper foil a practical and effective platform for the hydrogen-evolution reaction.

The present study investigates the synthesis of a CuFeO_2_/Cu_2_ZnSnS_4_/Cs_2_SnCl_6_ multilayer photoelectrode designed for solar-driven water splitting using wastewater as the feed solution. To the best of our knowledge, this work represents the first report on the fabrication and application of this ternary multilayer architecture for hydrogen generation. Extensive structural, optical, and electrochemical analyses were carried out to evaluate the properties and interfacial behavior of the stacked layers. The PEC performance was measured under varying light intensities and wavelengths to elucidate the reaction kinetics and overall conversion efficiency of the water-splitting process. Key thermodynamic parameters were also determined. The multilayer system achieved a high incident photon-to-current efficiency of 77%, confirming that the drop-casting method can deliver competitive performance while maintaining simplicity and scalability. Utilizing wastewater as a hydrogen source integrates renewable energy production with environmental remediation, thereby simultaneously addressing critical energy and water challenges. These results underscore the potential of this multifunctional, solid-state photoelectrode design for advancing sustainable technologies toward practical, real-world applications.

## 2. Experimental

The complete sets of essential chemicals employed in this work were procured from Sigma-Aldrich (USA) and were of high purity to ensure experimental accuracy and reliability. The specific reagents included dimethylformamide (99.8%), tin(ii) chloride (99.8%), cesium chloride (99.8%), toluene (99.0%), anhydrous methanol (99.8%), oleylamine (98.8%), carbon disulfide (99.8%), anhydrous chloroform (99.8%), tin(ii) acetate (95.5%), anhydrous anisole (99.9%), copper acetate (98.2%), dehydrated zinc acetate (99.8%), ethanol (99.9%), iron(ii) nitrate (99.8%), concentrated sulfuric acid (99.8%), and acetone (99.8%).

### 2.1 CuFeO_2_ synthesis

The CuFeO_2_ layer was prepared using a combustion method.^30,31^ Initially, high-purity copper (Cu) foil was subjected to ultrasonic cleaning for 20.0 minutes, first in sulfuric acid and subsequently in distilled water to remove surface oxides and impurities. This was followed by sequential ultrasonic cleaning in distilled water, acetone, and ethanol for 10.0 minutes each to ensure thorough surface preparation. After cleaning, the copper foil was immersed in a 0.2 M iron(ii) nitrate solution for 20.0 minutes. After soaking, the foil was dried at 70.0 °C for 40.0 minutes. Finally, thermal annealing was performed at 400 °C for 15 minutes in ambient conditions, resulting in the formation of a CuFeO_2_ layer on the Cu substrate.

### 2.2 Cu_2_ZnSnS_4_ synthesis

The second Cu_2_ZnSnS_4_ layer was synthesized using a straightforward and efficient hydrothermal procedure.^32^ In this process, 0.087 g of tin(ii) acetate, 0.34 g of copper(ii) acetate, and 0.061 g of zinc(ii) acetate were incorporated in 50.0 mL of methoxybenzene. The prepared solution was stirred and heated at 40 °C using a hot plate at 90 rpm, yielding a light blue solution. Subsequently, 1.20 mL of oleylamine was added, causing the solution to turn deep blue. Then, the process continued with the addition of 0.5 mL of carbon disulfide, which changed the color to a deep yellow. The solution was subsequently placed in a sealed autoclave lined with Teflon and underwent hydrothermal processing for 18 hours at 190 °C. After cooling to ambient temperature, a black precipitate was collected by centrifugation. A two-step washing process was employed to remove residual anisole, in which the precipitate was first washed with toluene and then with methanol, followed by centrifugation at 3000 rpm for 25 minutes after each step. Finally, 0.054 g of the purified Cu_2_ZnSnS_4_ powder was dissolved in 3.0 mL of chloroform to prepare the Cu_2_ZnSnS_4_ solution for layer deposition.

### 2.3 Cs_2_SnCl_6_ synthesis

To synthesize the Cs_2_SnCl_6_ perovskite layer, 4.0 mL of dimethylformamide was used as the solvent to dissolve tin(ii) chloride and cesium chloride in a 1 : 1 molar ratio. The solution was heated at 40 °C on a hot plate and stirred at 60 rpm for 1.5 hours, during which the mixture gradually transformed into a deep yellow color.

### 2.4 CuFeO_2_/Cu_2_ZnSnS_4_/Cs_2_SnCl_6_ synthesis

The CuFeO_2_/Cu_2_ZnSnS_4_/Cs_2_SnCl_6_ multilayer structures were fabricated using a drop-casting method. Compared with evaporation or sputtering techniques, drop-casting offers clear advantages for scalable material production. Its minimal equipment requirements and straightforward setup enable an easy transition from laboratory-scale preparation to pilot-scale manufacturing. Because the process is performed under ambient conditions, it reduces thermal and chemical stress, thereby preserving the structural and chemical integrity of the deposited materials. Furthermore, drop-casting facilitates rapid prototyping with short processing cycles and allows reliable control over film thickness and morphology through simple adjustments of solution concentration and deposition parameters.

The Cu_2_ZnSnS_4_ layer was deposited onto the Cu/CuFeO_2_ by drop-casting 110 µL of a Cu_2_ZnSnS_4_ solution at 80 °C for 20 minutes. This was followed by annealing the resulting Cu/CuFeO_2_/Cu_2_ZnSnS_4_ structure at 145 °C for 20 minutes. Subsequently, the CsSnCl perovskite layer was deposited by drop casting 110 µL of CsSnCl solution onto the CuFeO_2_/Cu_2_ZnSnS_4_ layer at 60 °C for 20 minutes. Finally, the complete CuFeO_2_/Cu_2_ZnSnS_4_/Cs_2_SnCl_6_ was annealed in air at 135 °C for 20 minutes.

### 2.5 Surface and structural characterization

The surficial structure properties of the CuFeO_2_/Cu_2_ZnSnS_4_/Cs_2_SnCl_6_ were analyzed through scanning electron microscopy (SEM) at 5 kV with an Auriga Zeiss FIB instrument. X-ray diffraction (XRD) analysis was conducted utilizing a D5000 diffractometer to investigate the crystal structure of the prepared layers. The chemical composition and elemental states of the layers were analyzed using X-ray photoelectron spectroscopy (XPS) with a K-Alpha system (Thermo Fisher Scientific). Optical properties were evaluated using a UV-vis spectrophotometer with a double-beam configuration (PerkinElmer Lambda 950).

### 2.6 Hydrogen production

The PEC performance was evaluated using an electrochemical workstation (CHI660E). The measurement setup is shown in Fig. S1 (SI file). A standard three-electrode configuration was employed, where the CuFeO_2_/Cu_2_ZnSnS_4_/Cs_2_SnCl_6_ film (1.0 cm^2^) served as the working electrode, Ag/AgCl functioned as the reference electrode, and a platinum sheet acted as the counter electrode. Third-stage treated sewage water collected from Beni-Suef City, Egypt, was used as the electrolyte. Illumination was supplied by a solar simulator equipped with a Newport xenon lamp delivering a light intensity of 100 mW cm^−2^. Since the reversible hydrogen electrode (RHE) is widely used in electrochemical studies. The potentials were originally recorded against the Ag/AgCl reference electrode. All measured potentials were converted to the RHE scale using the Nernst equation^33,34^

1 *V*(RHE) = *V*(Ag/AgCl) + 0.059 pH + 0.1976 V

here, pH refers to the value of the electrolyte used during the PEC measurements. This conversion ensures the accurate referencing of electrochemical data and enables a meaningful comparison with hydrogen evolution reaction benchmarks.

## 3. Results and discussion

### 3.1. SEM (scanning electron microscopy)

**Fig. 1:**
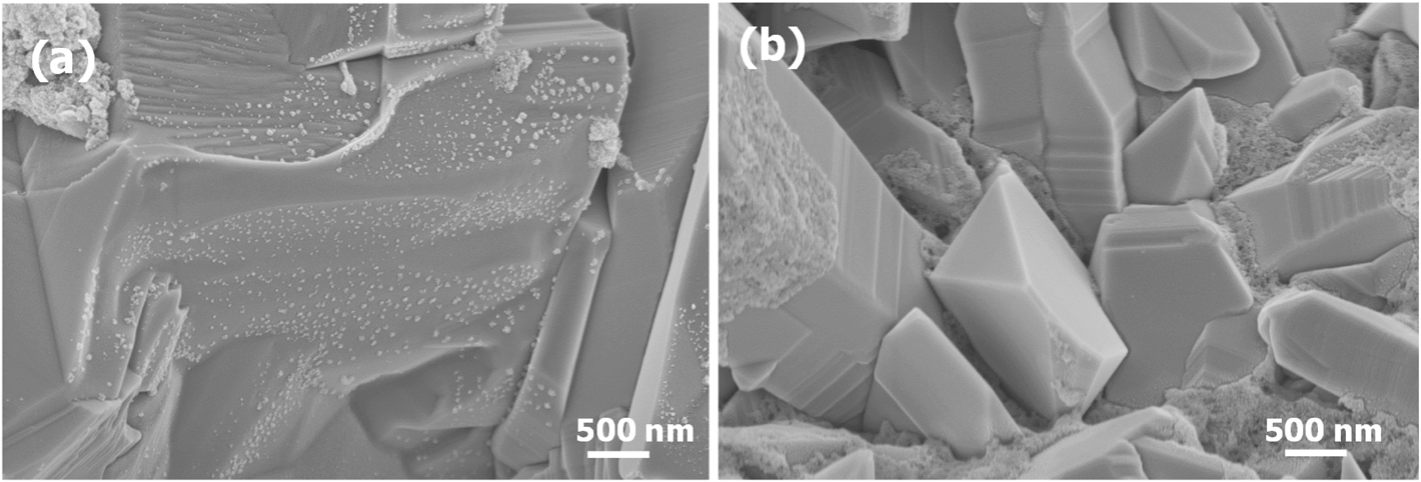
SEM images of (a) CuFeO_2_, (b) CuFeO_2_/Cu_2_ZnSnS_4_.

The surface morphologies of CuFeO_2_, CuFeO_2_/Cu_2_ZnSnS_4_, and CuFeO_2_/Cu_2_ZnSnS_4_/Cs_2_SnCl_6_ heterostructures were examined using SEM. The SEM image of CuFeO_2_ reveals a relatively smooth, plate-like morphology composed of stacked and overlapping lamellar structures, Fig. 1(a). These densely packed layers form a compact architecture that enhances the layer's structural integrity. Small, granular particles are distributed across the lamellar surfaces, introducing a degree of surface roughness that is beneficial for adhesion and stability in heterostructure configurations.

Upon Cu_2_ZnSnS_4_ deposition, a significant transformation in surface morphology is observed. Fig. 1(b) shows that the CuFeO_2_/Cu_2_ZnSnS_4_ multilayer exhibits well-faceted grains with pyramid-like or prismatic geometries. The lateral dimensions of these structures range from approximately 600 to 2100 nm. These densely packed grains exhibit a rougher, more granular texture than pure CuFeO_2_. The sharp edges, distinct grain boundaries, and clearly defined crystallographic facets confirm the polycrystalline nature of Cu_2_ZnSnS_4_ with high crystallinity. The Cu_2_ZnSnS_4_ layer also demonstrates a pore-free surface of compact, well-defined particles. In addition to the dominant faceted grains, some non-uniform particles and finer particulates are present. This morphological evolution confirms the successful deposition of Cu_2_ZnSnS_4_ onto CuFeO_2_ and the effective formation of a coherent heterostructure.

**Fig. 2:**
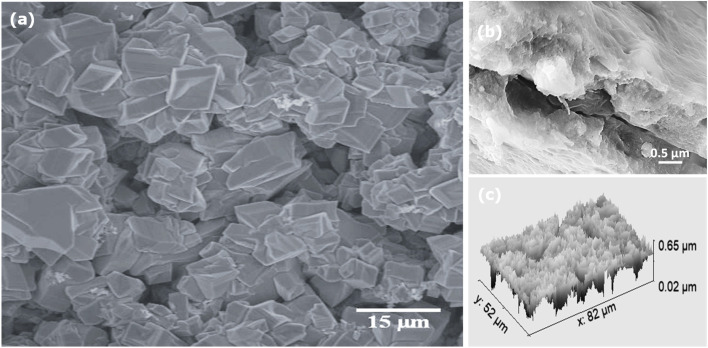
SEM analysis of the CuFeO_2_/Cu_2_ZnSnS_4_/Cs_2_SnCl_6_ multilayer structure: (a) top-view image, (b) cross-sectional image, and (c) 3D surface topography.

Fig. 2(a) presents SEM images of the CuFeO_2_/Cu_2_ZnSnS_4_/Cs_2_SnCl_6_ multilayered ternary heterostructure. The CuFeO_2_/Cu_2_ZnSnS_4_ bilayer is uniformly coated with Cs_2_SnCl_6_ perovskite, producing a smooth surface free of holes or major cracks, which indicates high structural stability. The surface exhibits a homogeneous distribution of nanocrystal-like features with an average grain size of approximately 6.1 µm. These nanocrystals are densely packed and interconnected, forming a continuous and adherent layer. Their angular, well-faceted morphology and compact arrangement confirm the successful formation of polycrystalline Cs_2_SnCl_6_. The closely packed nanocrystalline network generates a pore-like microstructure, advantageous for PEC applications as it enhances light scattering and facilitates electrolyte penetration into the photoelectrode.

Fig. 2(b) presents the cross-sectional SEM image of the CuFeO_2_/Cu_2_ZnSnS_4_/Cs_2_SnCl_6_ multilayer structure, revealing a total thickness of approximately 400 nm. Fig. 2(c) shows a 3D topographical view of the surface. The surface texture parameters were quantitatively evaluated using the open-source Gwyddion software. The mean surface roughness (*S*_a_) was 58.57 nm, while the root mean square roughness (*S*_q_) was slightly higher at 80.49 nm, indicating a high rough nanoscale morphology. This degree of roughness is beneficial for PEC applications, as it enhances light scattering and increases the electrochemically active surface area (ECSA), thereby improving photon absorption and boosting the density of catalytic sites for redox reactions. The observed roughness results from multi-phase integration and hierarchical grain stacking, which together contribute to the complex surface architecture. The skewness (*S*_sk_) value of −1.145 indicates a surface dominated by valleys rather than peaks. Such negative skewness facilitates electrolyte wetting, promotes reactant retention near active regions, and enhances reaction kinetics. Deep valleys further support electrolyte penetration, ensuring sustained interfacial contact during PEC operation. The excess kurtosis (*S*_ku_) value of 1.887 reflects a leptokurtic height distribution, characterized by sharp features and frequent extreme deviations. Additional insight into vertical relief is provided by the maximum peak height (*S*_p_) and maximum pit depth (*S*_v_), measured at 212.0 nm and 415.4 nm, respectively. These values suggest an extended optical path length through enhanced light scattering. Hence, the SEM morphology confirms the successful formation of heterojunctions between CuFeO_2_, Cu_2_ZnSnS_4_, and Cs_2_SnCl_6_ with improved PEC performance.

### 3.2. XRD analysis

The crystal structures of the synthesized CuFeO_2_, CuFeO_2_/Cu_2_ZnSnS_4_, and CuFeO_2_/Cu_2_ZnSnS_4_/Cs_2_SnCl_6_ heterostructures were analyzed using X-ray diffraction (XRD).

**Fig. 3:**
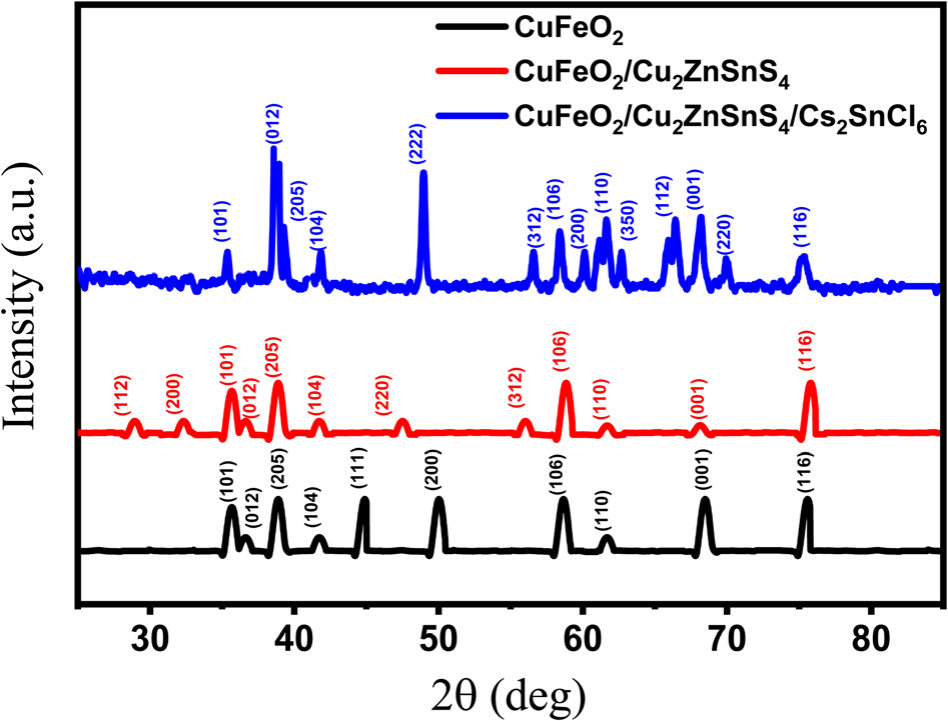
XRD diagram of CuFeO_2_, CuFeO_2_/Cu_2_ZnSnS_4_, and CuFeO_2_/Cu_2_ZnSnS_4_/Cs_2_SnCl_6_ multilyers.

Fig. 3 (black curve) illustrates the X-ray diffraction pattern of the CuFeO_2_ layer, revealing the coexistence of two crystallographic phases, rhombohedral 3R CuFeO_2_ and hexagonal 2H CuFeO_2_, corresponding to JCPDS card numbers 000390246 and 010791546, respectively.^18,35^ The rhombohedral (3R) phase of CuFeO_2_ is dominant, as indicated by the higher intensity of its characteristic peaks. The diffraction peaks at 35.06°, 36.42°, 39.26°, 42.0°, 58.42°, 61.64°, 68.24°, and 75.32° are indexed to the (101), (012), (205), (104), (106), (110), (001), and (116) planes of CuFeO_2_, respectively. The mean size of the crystallites determined through the Scherrer equation is approximately 67.0 nm. Minor peaks at 44.72° and 50.0°, corresponding to the (111) and (200) planes, confirm the presence of trace CuO secondary phases. These results demonstrate the successful crystallization of CuFeO_2_ with minor CuO inclusions.

Fig. 3 (red color) shows the XRD pattern of the CuFeO_2_/Cu_2_ZnSnS_4_ bilayer. Peaks at 28.29°, 32.2°, 47.84°, and 56.08° match the kesterite phase at (112), (200), (220), and (312) crystal planes of the Cu_2_ZnSnS_4_ crystal planes in agreement with JCPDS card number 26-0575.^36^ This confirms its successful crystallization of Cu_2_ZnSnS_4_ on the CuFeO_2_ layer.^37^ The calculated crystallite size of Cu_2_ZnSnS_4_ is approximately 45.6 nm. Additional reflections at 35.06°, 36.42°, 39.26°, 42.0°, 58.42°, 61.64°, 68.24°, and 75.32° correspond to the (101), (012), (205), (104), (106), (110), (001), and (116) planes of CuFeO_2_. The results are consistent with previously reported findings on kesterite Cu_2_ZnSnS_4_.^37^ The coexistence of CuFeO_2_ and Cu_2_ZnSnS_4_ phases indicates high crystallinity and excellent structural compatibility, which are essential for efficient heterostructure formation and charge transfer in PEC systems.

Fig. 3 (blue color) presents the XRD pattern of the CuFeO_2_/Cu_2_ZnSnS_4_/Cs_2_SnCl_6_ ternary heterostructure. The observed diffraction peaks confirm the formation of cubic-phase Cs_2_SnCl_6_ (space group *Fm*3*m*), consistent with JCPDS card number 75-0376.^38,39^ Reflections at 38.96°, 60.14°, 66.29°, and 69.94°, respectively, match the (012), (200), (112), and (220) crystal planes. The (012) peak exhibits the highest intensity, indicating preferential orientation. The calculated crystallite size for Cs_2_SnCl_6_ is approximately 17.74 nm. Peaks at 35.38°, 39.26°, 42.0°, 56.58°, 58.42°, 61.64°, 68.24°, and 75.32° correspond to CuFeO_2_/Cu_2_ZnSnS_4_ bilayer phases, confirming successful integration of all three materials. The distinct and narrow diffraction peaks, along with the absence of impurity forms, indicate a low dislocation density and good crystallinity, which are crucial for enhancing charge carrier mobility, minimizing recombination losses, and improving the efficiency of the PEC water-splitting system.

The graded crystallite sizes within the multilayer heterostructure establish a hierarchical morphology, producing a synergistic enhancement of hydrogen production. The nanoscale crystallite size of the Cs_2_SnCl_6_ layer (17.74 nm) promotes strong photon absorption through enhanced light scattering, providing a high surface-to-volume ratio that accelerates interfacial charge transfer to the electrolyte. The intermediate crystallite size of the Cu_2_ZnSnS_4_ layer (45.6 nm) ensures balanced charge transport and efficient light harvesting, facilitating effective electron–hole separation across the heterojunction. It can provide potential active catalytic sites, though grain boundaries may trap carriers. In contrast, the larger crystallite size of the CuFeO_2_ layer (67.0 nm) reduces grain boundary density, enabling improved carrier mobility, deeper photon penetration, and minimized recombination losses.

### 3.3. XPS (X-ray photoelectron spectra)

**Fig. 4:**
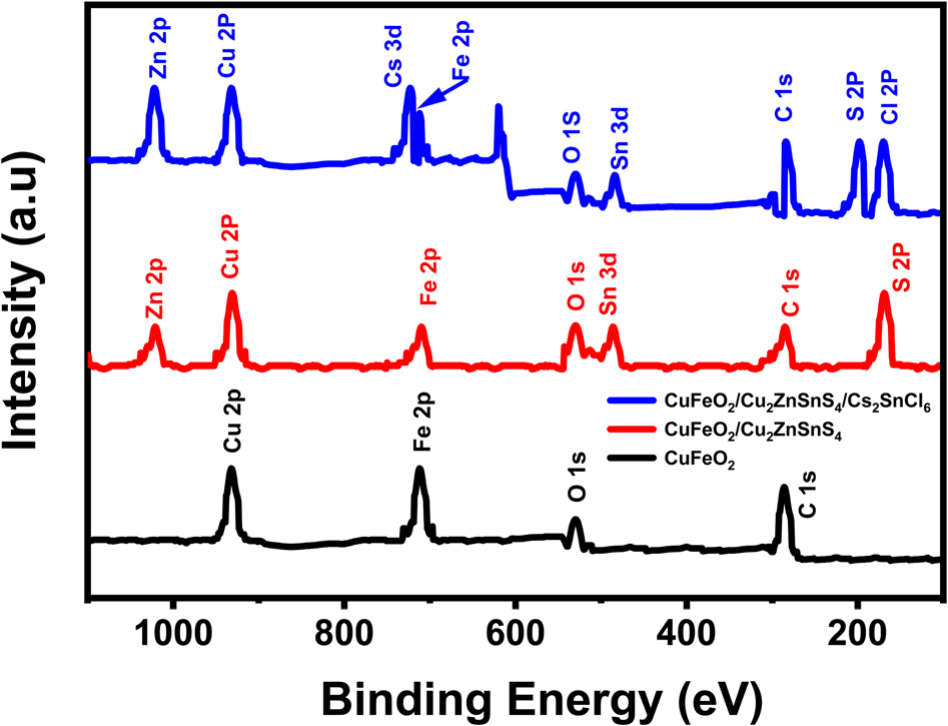
XPS of CuFeO_2_, CuFeO_2_/Cu_2_ZnSnS_4_, and CuFeO_2_/Cu_2_ZnSnS_4_/Cs_2_SnCl_6_ multilayers.

Surface chemical structures and elemental states of the synthesized CuFeO_2_, CuFeO_2_/Cu_2_ZnSnS_4_, and CuFeO_2_/Cu_2_ZnSnS_4_/Cs_2_SnCl_6_ heterostructures were analyzed using XPS. The full survey spectra for these layers are shown in Fig. 4.

Fig. 4 (black color) presents the XPS survey spectrum of the CuFeO_2_ layer, showing characteristic core-level peaks corresponding to O 1s, Fe 2p, C 1s, and Cu 2p at binding energies (BEs) of, respectively, 530.98, 711.25, 285.0, and 932.61 eV. All the above results confirm the successful formation of the CuFeO_2_ phase *via* the combustion synthesis method.

Fig. 4 (red color) illustrates the XPS survey spectrum of the CuFeO_2_/Cu_2_ZnSnS_4_ bilayer structure. Prominent peaks are observed at 711.25 eV (Fe 2p), 1022.06 eV (Zn 2p), 530.98 eV (O 1s), 485.75 eV (Sn 3d), 932.71 eV (Cu 2p), 168.34 eV (S 2p), and 285.0 eV (C 1s). The presence of these elements verifies the successful deposition of Cu_2_ZnSnS_4_ onto the CuFeO_2_ layer and confirms the formation of the bilayer heterostructure. Fig. 4 (blue color) displays the XPS survey spectrum of the CuFeO_2_/Cu_2_ZnSnS_4_/Cs_2_SnCl_6_ multilayer. Distinct peaks associated with Cs, Cl, Fe, Sn, S, Zn, and Cu are detected, confirming the incorporation of the Cs_2_SnCl_6_ layer into the underlying CuFeO_2_/Cu_2_ZnSnS_4_ bilayer and the successful construction of the final ternary heterostructure. Additionally, minor peaks related to C 1s and O 1s are observed, which are attributed to the physisorption of atmospheric oxygen and carbon species during air exposure.^40^

Fig. S2 (SI file) provides a detailed high-resolution XPS analysis of the CuFeO_2_/Cu_2_ZnSnS_4_/Cs_2_SnCl_6_ heterostructure, offering insight into the chemical states of its constituent elements. In Fig. S2(a), the Cs 3d core-level spectrum exhibits two distinct peaks at 737.8 eV and 723.9 eV, corresponding to Cs 3d_3/2_ and Cs 3d_5/2_, confirming the presence of Cs^+^ ions.^41^ Fig. S2(b) shows the Sn 3d spectrum peaks that appeared at 494.0 eV for Sn 3d_3/2_ and 485.6 eV for Sn 3d_5/2_.^42^ From Fig. S2(c), the Cl 2p spectrum displays symmetric Gaussian peaks for Cl 2p_3/2_ and Cl 2p_1/2_ at 197.1 eV and 198.5 eV, confirming Cl^−^ ions in the structure.^43^ The Cu 2p spectrum in Fig. S2(d) shows Cu 2p_1/2_ and Cu 2p_3/2_ peaks at 952.0 eV and 932.0 eV, verifying the Cu(i) state in CuFeO_2_.^37^ Fig. S2(e) presents peaks at 1045.0 eV and 1022.0 eV for Zn 2p_1/2_ and Zn 2p_3/2_, corresponding to Zn^2+^ in Cu_2_ZnSnS_4_.^44^ The S 2p spectrum in Fig. S2(f) reveals two peaks: one between 168.0 and 172.0 eV associated with S^6+^, and another between 160.0 and 164.0 eV corresponding to elemental sulfur (S^0^), indicating partial surface oxidation.^37^ Fig. S2(g) displays the Fe 2p_3/2_ peak at 711.0 eV and accompanying satellite peaks near 713.6 eV, confirming the presence of Fe^3+^ species.^45^ The C 1 s spectrum in Fig. S2(h) shows a 285.0 eV peak attributed to C–C bonding, likely from surface-adsorbed hydrocarbons.^46^ Lastly, the O 1s spectrum in Fig. S2(i) shows a peak around 530.0 eV, corresponding to lattice O^2−^ ions. Collectively, all key elements are present in their expected oxidation states. These XPS results confirm the successful formation and chemical integrity of the CuFeO_2_/Cu_2_ZnSnS_4_/Cs_2_SnCl_6_ multilayer.

### 3.4. Optical analysis

**Fig. 5:**
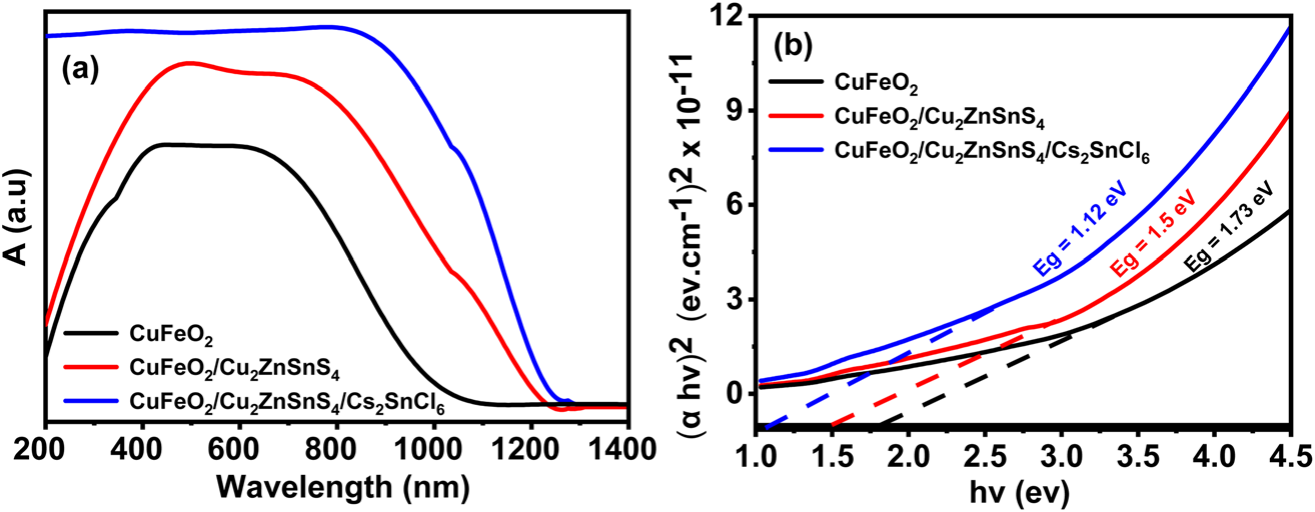
(a) Absorption spectrum and (b) Tauc plot of CuFeO_2_, CuFeO_2_/Cu_2_ZnSnS_4_, and CuFeO_2_/Cu_2_ZnSnS_4_/Cs_2_SnCl_6_.

The optical properties of photocatalysts are critical for assessing their performance in PEC applications. Fig. 5(a) presents the absorption spectra of the fabricated samples across the UV-vis-NIR range: CuFeO_2_ (black curve), CuFeO_2_/Cu_2_ZnSnS_4_ (red curve), and CuFeO_2_/Cu_2_ZnSnS_4_/Cs_2_SnCl_6_ (blue curve). All three samples exhibit broad absorption over a wide spectral range (400–800 nm), indicating their potential for solar energy harvesting. The bare CuFeO_2_ layer shows the weakest absorption, primarily in the visible region, indicating limited light-harvesting capability. Incorporating Cu_2_ZnSnS_4_ into CuFeO_2_ to form a binary heterostructure significantly enhances the absorption intensity and extends the range into the near-infrared region.

Among the three configurations, the ternary CuFeO_2_/Cu_2_ZnSnS_4_/Cs_2_SnCl_6_ heterostructure demonstrates the strongest and broadest absorption, confirming its superior ability to utilize a wider portion of the solar spectrum. This improvement can be explained by the synergistic integration of multiple semiconductors with complementary optical properties. Specifically, Cu_2_ZnSnS_4_ and Cs_2_SnCl_6_ introduce additional light-absorbing states, broadening the spectral response and improving photon absorption. This increased absorption facilitates more efficient photoexcitation and charge carrier generation under solar illumination, which is crucial for enhancing PEC activity, particularly in hydrogen evolution reactions.

Fig. 5(b) presents the Tauc plots for estimating the optical band gaps of the three prepared layers. The direct allowed transitions are given by Tauc's equation.^47,48^

2 *K*(*νh* − *E*_g_)^1/2^ = *ανh*

where *K* is a constant, *ν* means light frequency, *h* refers Planck's constant, and *α* is the absorption coefficient.

The absorption coefficient *α* was calculated from the absorbance (*A*) and layer thickness (*d*) using the relation

3 *α* = 2.303*A*/*d*

The optical band gaps were derived by extending the linear section of the (*αhν*)^2^*versus hν* curves to intersect the *x*-axis. The measured band-gap values are 1.73 eV related to CuFeO_2_, 1.50 eV belong to CuFeO_2_/Cu_2_ZnSnS_4_, and 1.12 eV associated with CuFeO_2_/Cu_2_ZnSnS_4_/Cs_2_SnCl_6_. The gradual narrowing of the band gap with the addition of Cu_2_ZnSnS_4_ and Cs_2_SnCl_6_ suggests enhanced light absorption and improved carrier excitation. Hence, the ternary CuFeO_2_/Cu_2_ZnSnS_4_/Cs_2_SnCl_6_ heterostructure exhibits the lowest band gap and the highest solar absorption.

### 3.5. Photoelectrochemical H_2_ generation

#### 3.5.1. Effect of dark, light, and stability

**Fig. 6:**
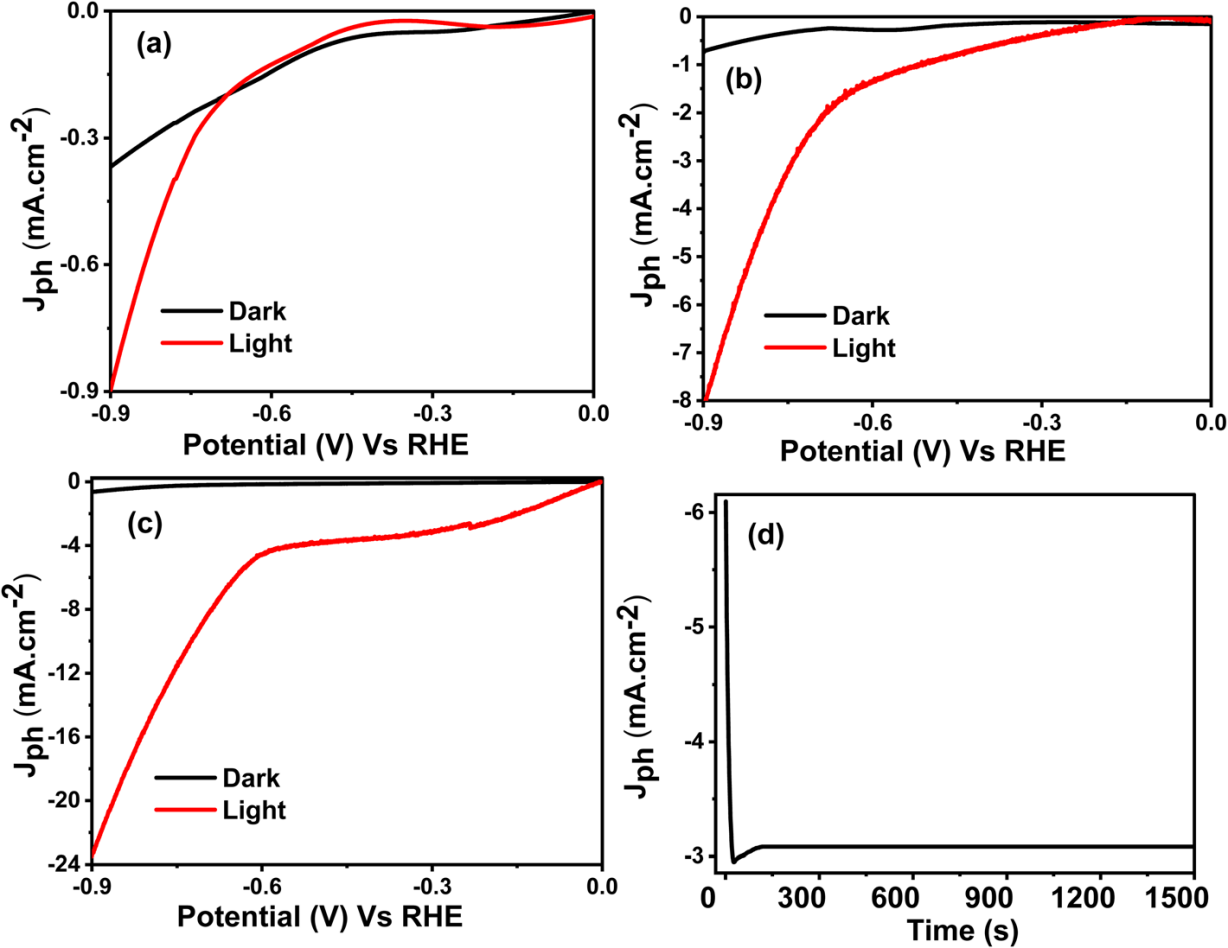
PEC processes (a) CuFeO_2_, (b) CuFeO_2_/Cu_2_ZnSnS_4_, (c) CuFeO_2_/Cu_2_ZnSnS_4_/Cs_2_SnCl_6_ (d) current–time characteristic of CuFeO_2_/Cu_2_ZnSnS_4_/Cs_2_SnCl_6_.

Fig. 6(a–c) illustrates the PEC response of CuFeO_2_, CuFeO_2_/Cu_2_ZnSnS_4_, and CuFeO_2_/Cu_2_ZnSnS_4_/Cs_2_SnCl_6_ multilayer photoelectrodes. The PEC procedures were performed at ambient conditions using a wastewater solution reflecting practical and environmentally relevant operating conditions. A linear voltage sweep rate of 20.0 mV s^−1^ is applied in both dark and visible light environments. An intensity of 100 mW cm^−2^ is applied utilizing a Xenon illumination lamp. This allows for a comprehensive analysis of the electrochemical behavior and light-induced activity of photoelectrodes.

As the applied potential increased, a corresponding rise in current was observed, which can be attributed to enhanced charge transport through tunneling mechanisms at the electrode–electrolyte interface.^49^ Under dark conditions, all electrodes exhibited relatively low current densities due to the absence of photogenerated carriers. The recorded dark current densities were − 0.37 mA cm^−2^ for CuFeO_2_, −0.60 mA cm^−2^ for CuFeO_2_/Cu_2_ZnSnS_4_, and −0.31 mA cm^−2^ for CuFeO_2_/Cu_2_ZnSnS_4_/Cs_2_SnCl_6_. These currents primarily result from ionic charge transfer through the electrolyte.^50^

Upon illumination, all samples showed a notable enhancement in photocurrent density, indicating efficient generation and separation of electron–hole pairs. The photocurrent densities under light were significantly enhanced, reaching −0.9 mA cm^−2^ for CuFeO_2_, −8.0 mA cm^−2^ related to CuFeO_2_/Cu_2_ZnSnS_4_, and −24.0 mA cm^−2^ associated with CuFeO_2_/Cu_2_ZnSnS_4_/Cs_2_SnCl_6_. This apparent improvement in photocurrent response confirms the contribution of photogenerated carriers to the PEC process and reflects better photoactivity under solar-like conditions.

The progressive enhancement in photocurrent from the monolayer CuFeO_2_ to the binary CuFeO_2_/Cu_2_ZnSnS_4_ and finally to the ternary CuFeO_2_/Cu_2_ZnSnS_4_/Cs_2_SnCl_6_ structure highlights the impact of compositional engineering on PEC efficiency. The construction of multilayer heterostructures significantly enhances PEC performance by promoting improved charge carrier dynamics and more efficient light harvesting.

The superior performance of the CuFeO_2_/Cu_2_ZnSnS_4_/Cs_2_SnCl_6_ heterostructure is attributed to several synergistic effects, including enhanced optical absorption, a narrower band gap, improved crystallinity, and suppressed recombination of charge carriers. Notably, forming internal electric fields at the interfaces between the semiconductors, resulting from the favorable arrangement of energy bands, is vital for facilitating directional charge separation and migration.^51^ This reduces recombination losses and supports sustained carrier flow toward the reaction sites. Therefore, this ternary heterostructure offers a promising approach for efficient solar-driven hydrogen generation, especially when utilizing wastewater as a practical and sustainable resource.

The complex composition of sewage water has a direct impact on PEC hydrogen production. Organic pollutants (phenols, pesticides, surfactants) can act as sacrificial hole scavengers, reducing electron–hole recombination and supporting higher photocurrent generation. Inorganic ions (Na^+^, K^+^, Mg^+2^, Ca^2+^) improve ionic conductivity and lower solution resistance, while transition-metal ions (Fe^3+^, Co^2+^, Mn^2+^, Cu^2+^) may participate in redox cycling that promotes interfacial electron transfer. Trace amounts of heavy-metal ions (Hg^2+^, Pb^2+^, Cd^3+^, Cr^3+^) can also alter surface states in ways that facilitate charge transport. Nutrient ions (NH_4_^+^, PO_4_^3−^) influence charge dynamics by modifying the local interfacial environment. At elevated concentrations, however, these same species can become detrimental, leading to surface fouling, additional recombination pathways, and enhanced photocorrosion. Hence, the use of sewage water provides a dual benefit. It supports high hydrogen evolution for clean energy production while simultaneously enabling the degradation of pollutants – addressing key environmental challenges.

Fig. 6(d) shows the chronoamperometric stability test of the CuFeO_2_/Cu_2_ZnSnS_4_/Cs_2_SnCl_6_ photoelectrode under continuous illumination. This test is crucial for evaluating the practical applicability of the electrode, particularly in systems designed for sustainable hydrogen production that utilize wastewater as the electrolyte. At the beginning of the measurement, a sharp decline in photocurrent density (*J*_ph_) is observed, dropping rapidly from −6.1 to −2.95 mA cm^−2^. Such a decline is typical in PEC systems and is generally attributed to transient reactions at the electrode–electrolyte boundary.^52^ This initial drop is likely due to surface reactions between the electrode and reactive species in the wastewater. These reactions may include mild surface oxidation or temporary blockage of active sites, which can hinder charge carrier transfer. Such phenomena are commonly associated with early-stage corrosion or imbalances in charge accumulation, particularly in complex aqueous environments, such as wastewater.

After this initial phase, the *J*_ph_ stabilizes at approximately −3.13 mA cm^−2^ throughout the test, indicating that the electrode surface has adapted and that charge transfer kinetics have improved. This stable current response demonstrates excellent operational durability, minimal photodegradation, and strong chemical resistance of the ternary photoelectrode. The long-term stability of the CuFeO_2_/Cu_2_ZnSnS_4_/Cs_2_SnCl_6_ heterostructure during prolonged water-splitting operation can be attributed to several factors. Its layered architecture provides a favorable arrangement of energy bands, facilitating the development of a coherent heterostructure. Additionally, the surface morphology supports improved charge dynamics. Internal electric fields generated at the heterojunction interfaces facilitate the movement of photogenerated charge carriers toward reaction sites, thereby minimizing recombination losses. Furthermore, the low dislocation density and high crystallinity contribute to enhanced resistance against corrosion.

Using Faraday's law of electrolysis, the rate of H_2_ generation was calculated to be 0.039 mA h^−1^ under real wastewater conditions. The combination of high photocurrent response, excellent operational stability, and resilience in harsh electrolytes highlights the strong potential of this ternary heterostructure for practical, solar-driven photoelectrochemical water-splitting applications.

#### 3.5.2. Monochromatic light and light power density effects

**Fig. 7:**
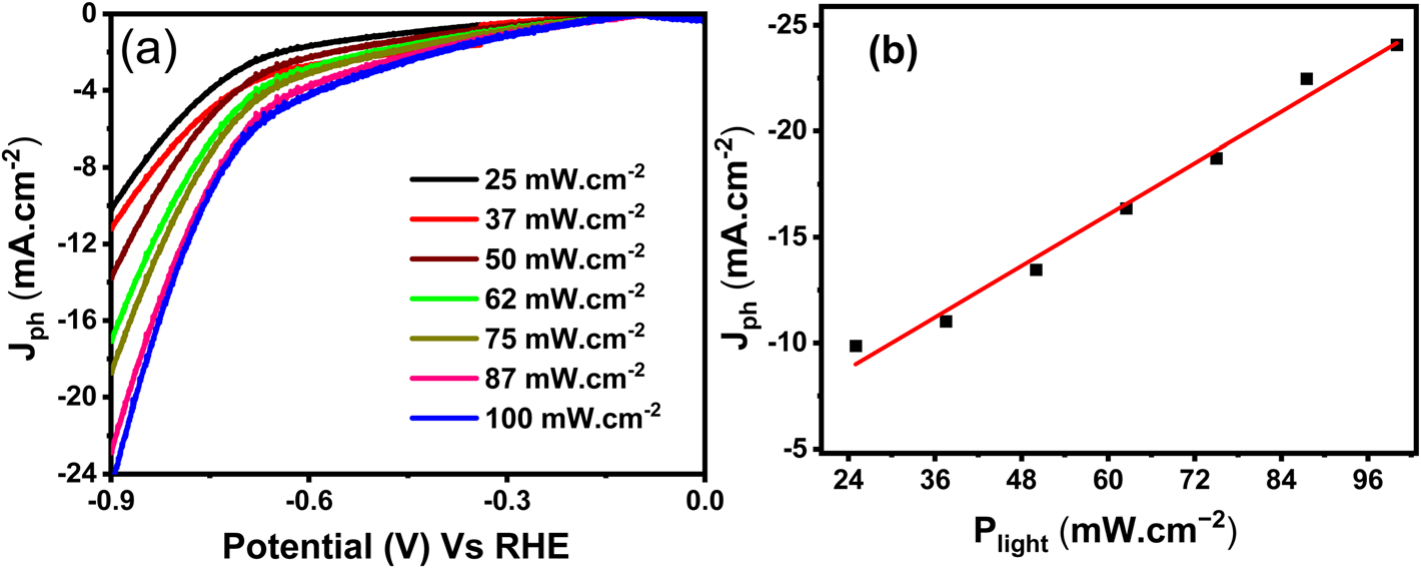
(a) *J*_ph_–*V* curve at several light power densities and (b) *J*_ph_–*P*_light_ curve for CuFeO_2_/Cu_2_ZnSnS_4_/Cs_2_SnCl_6_.

Fig. 7(a) presents the *J*_ph_–*V* curves for the ternary CuFeO_2_/Cu_2_ZnSnS_4_/Cs_2_SnCl_6_ photoelectrode under varying light power densities ranging from 25.0 to 100.0 mW cm^−2^. The *J*_ph_ increases consistently with higher light intensities across the tested voltage range. The shape of the *J*_ph_–*V* curves confirms a direct correlation between the photon flux (incoming light intensity) and the rate of electron–hole pair generation. Also, it reflects effective charge separation, efficient light absorption, and reduced charge transfer resistance at the boundary between the electrode and the electrolyte under higher light intensities, thereby facilitating improved carrier mobility and enhancing PEC performance. Fig. 7(b) illustrates the variation in photocurrent density (*J*_ph_) as a function of light power density (*P*_light_) at 1.0 V. The plot presents an increase in *J*_ph_ from 9.88 mA cm^−2^ (at 25.0 mW cm^−2^) to 22.47 mA cm^−2^ (at 100.0 mW cm^−2^). Overall, all results confirm that the CuFeO_2_/Cu_2_ZnSnS_4_/Cs_2_SnCl_6_ photoelectrode exhibits excellent photoresponsivity and efficient charge dynamics across a broad spectrum of illumination intensities.

**Fig. 8:**
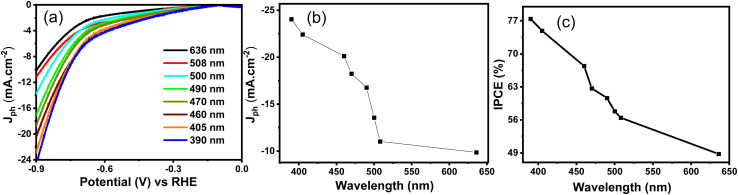
For the CuFeO_2_/Cu_2_ZnSnS_4_/Cs_2_SnCl_6_ (a) *J*_ph_–*V* curve at various monochromatic lights, (b) *J*_ph_–*λ* curve, and (c) IPCE curve.

Fig. 8 comprehensively evaluates the PEC behavior of the CuFeO_2_/Cu_2_ZnSnS_4_/Cs_2_SnCl_6_ photoelectrode under different monochromatic wavelengths, providing insight into its photon-to-electron conversion efficiency. Fig. 8(a) displays the *J*_ph_–*V* curve of the ternary heterostructure recorded under monochromatic light ranging from 390 to 636 nm. The photocurrent density (*J*_ph_) results reduce as the optical wavelength increases. The highest photocurrent is observed under 390 nm illumination, with a maximum *J*_ph_ of approximately −24.0 mA cm^−2^ (at −1.0 V), as shown in Fig. 8(b). This behavior indicates that the heterostructure is more responsive in the UV region. Higher-energy photons associated with shorter wavelengths make them more effective at exciting electrons across the band gap of the heterostructure, thereby enhancing the generation of electron–hole pairs. As the photon energy decreases with increasing wavelength, the carrier generation efficiency in the visible region becomes less pronounced.

Fig. 8(c) presents the wavelength-dependent incident photon-to-current conversion efficiency (IPCE). These measurements offer insight into the photoelectrode's ability to convert incident photons into a usable charge carrier. Eqn (4) calculates the IPCE at 1.0 V^53^4
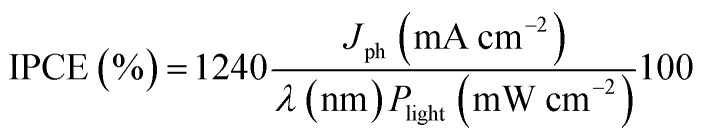
*J*_ph_ represents the photocurrent density measured at a specific wavelength, *λ* refers to the photon's wavelength, and *P*_light_ denotes the power density.

The IPCE curve confirms a high quantum efficiency, with the maximum value reaching approximately 77.0% at 390 nm. The efficiency gradually decreases with increasing wavelength, falling below 49.0% at 636 nm. These significant IPCE values indicate that the prepared CuFeO_2_/Cu_2_ZnSnS_4_/Cs_2_SnCl_6_ possesses excellent light-harvesting capability and is highly efficient in utilizing a broad region of the solar spectrum for hydrogen production.

#### 3.5.3. Temperature effect

**Fig. 9:**
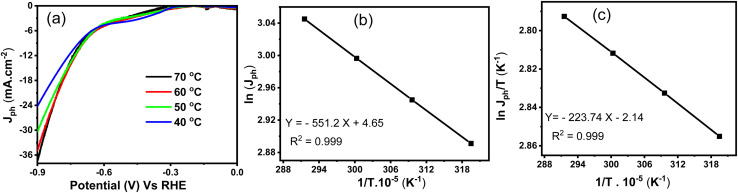
For the CuFeO_2_/Cu_2_ZnSnS_4_/Cs_2_SnCl_6_ (a) is the *J*_ph_–*V* relation at different temperatures, (b) is the temperature reciprocal and current density curve, and (c) represents the inverse of temperature and current density to temperature variation.

The temperature-dependent hydrogen generation performance of the CuFeO_2_/Cu_2_ZnSnS_4_/Cs_2_SnCl_6_ photoelectrode is illustrated in Fig. 9. This thermal behavior offers valuable insights into the charge transfer mechanisms underlying PEC activity. Fig. 9(a) presents the *J*_ph_–*V* curves recorded at 40 to 70 °C temperatures. A clear enhancement in photocurrent density is observed with increasing temperature, where *J*_ph_ rises from −24.0 to −36.5 mA cm^−2^. This increase is attributed to several interrelated physical and chemical factors that enhance the photoelectrode system's efficiency.^54,55^ Elevated thermal energy enhances charge carrier mobility and electrical conductivity within the semiconductor. The internal electric field strength also increases, promoting the successful separation of electrons and holes.

Additionally, higher temperatures decrease the potential barrier at the semiconductor/electrolyte interface, facilitating faster charge transfer. Temperature also accelerates surface reactions such as water oxidation and proton reduction, while improving ion diffusion in the electrolyte due to reduced viscosity. Furthermore, thermal excitation enables carriers to transition from sub-bandgap or defect states into the conduction band, increasing the carrier population available for photocurrent generation. Therefore, the photocurrent density (*J*_ph_) is a helpful indicator of the hydrogen generation rate under different thermal conditions. To further understand the kinetics, the activation energy (*E*_a_) is obtained from the Arrhenius equation, as expressed in eqn (5)

5 *k* = *A* e^−(*E*_a_/*TR*)^

here, *T* refers to the absolute temperature, *R* is the constant of the universal gas in Kelvin, *A* is related to the pre-exponential factor, and *k* is associated with the rate constant. The relationship between ln(*J*_ph_) and 1/*T*, shown in Fig. 9(b), exhibits a clear linear trend. The slope of the fitted line is −551.2, from which the calculated activation energy is 6.174 kJ mol^−1^. This result confirms that the CuFeO_2_/Cu_2_ZnSnS_4_/Cs_2_SnCl_6_ system exhibits thermally activated behavior, which is suitable for efficient hydrogen production.

Additionally, the Eyring equation is used to estimate thermodynamic parameters, including the activation enthalpy (ΔH*) and entropy (ΔS*), as described in eqn (6)6
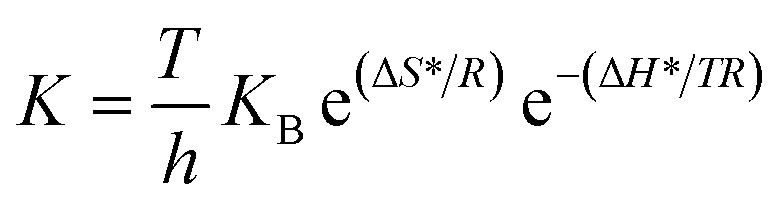
where ΔH* means the enthalpy change of the transition state, ΔS* refers to the change in the entropy, *h* is related to Planck's constant, and *K*_B_ is Boltzmann's constant. Fig. 9(c) shows the correlation of ln(*J*_ph_/*T*) and the inverse of temperature (1/*T*), which also demonstrates linearity. From the slope and intercept of the fit, ΔH* and ΔS* are determined to be 3.452 kJ mol^−1^ and 9.644 J mol^−1^ K^−1^, respectively. The low activation energy and favorable enthalpic and entropic parameters suggest that this heterostructure effectively utilizes thermal energy in conjunction with light, highlighting its potential for practical solar-driven hydrogen evolution applications.

#### 3.5.4. Impedance analysis

**Fig. 10:**
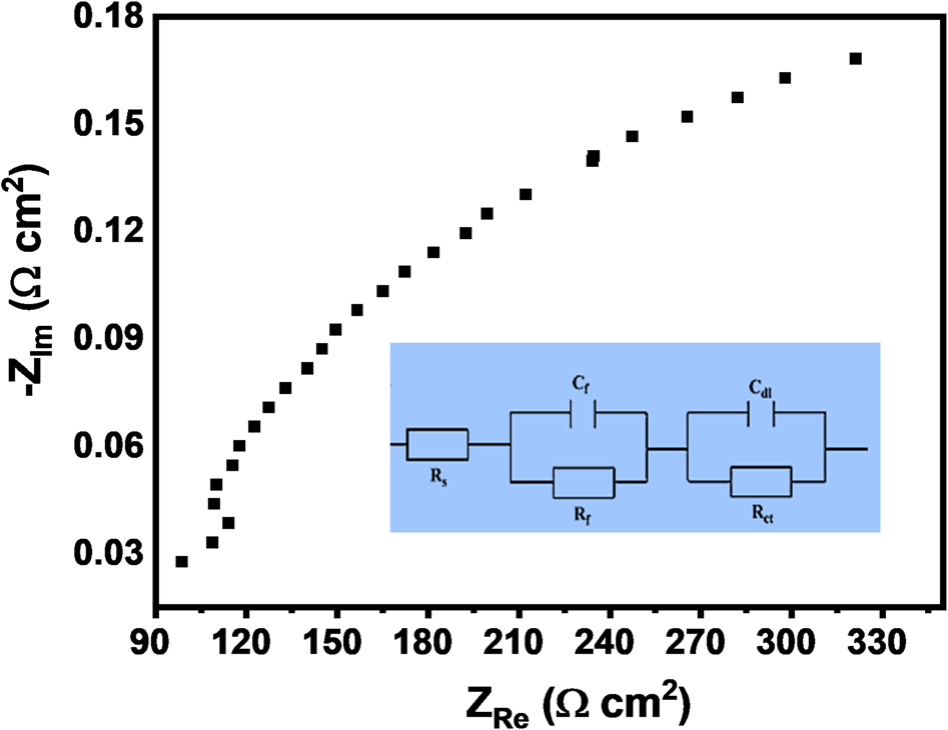
Nyquist impedance plot under white-light illumination with the inset showing the Randles equivalent circuit of the CuFeO_2_/Cu_2_ZnSnS_4_/Cs_2_SnCl_6_ multilayer structure.

Electrochemical impedance spectroscopy (EIS) provides valuable insights into the dynamics of electrochemical processes and the efficiency of charge transport within the photoelectrode system.^56–59^Fig. 10 presents the Nyquist impedance analysis of the CuFeO_2_/Cu_2_ZnSnS_4_/Cs_2_SnCl_6_ heterostructure under white light illumination. In the Nyquist plot shown in Fig. 10, the real part of the impedance (*Z*′) is plotted against the imaginary part (*Z*″), revealing two distinct semicircles that correspond to different charge transfer mechanisms occurring within the system. The smaller diameters of these semicircles indicate lower resistance and, therefore, better electrical conductivity. The high-frequency semicircles suggest rapid charge transfer, while the low-frequency region reflects the slower diffusion of charge carriers.^60^

The inset of Fig. 10 displays the fitted equivalent circuit model to interpret the impedance data. This model includes key elements such as *R*_s_. This solution resistance accounts for contributions from the electrolyte and electrode contacts, and *R*_ct_, representing the resistance to charge transfer at the boundary. Additional components, *R*_f_ and *C*_f_, model the resistance and capacitance associated with the charge separation and recombination dynamics. The double-layer capacitance (*C*_dl_) represents the capacitive properties of the electrical double layer at the electrode–electrolyte boundary. Combined with the high photocurrent density, these characteristics highlight the superior PEC performance of the CuFeO_2_/Cu_2_ZnSnS_4_/Cs_2_SnCl_6_ photoelectrode, rendering it a potential candidate for generating hydrogen from wastewater.

### 3.6. Mechanism

The Cs_2_SnCl_6_/Cu_2_ZnSnS_4_/CuFeO_2_ multilayer heterojunction combines complementary optical, electronic, and catalytic functions that together enhance hydrogen-evolution efficiency. The stacked architecture establishes a built-in electric field *via* graded band offsets. Under illumination, this internal field drives electrons upward toward the Cs_2_SnCl_6_/electrolyte interface and directs holes downward toward the CuFeO_2_ layer and the back contact. The resulting directional carrier flow improves charge-carrier transport pathways and effectively suppresses recombination.^61,62^ The electrons are transferred to the sewage water-electrolyte; these hot electrons cause the generation of *J*_ph_.^63^

The CuFeO_2_ layer serves primarily as the hole-collection layer. It is a p-type delafossite oxide that exhibits high hole mobility and an appropriate onset potential. The band alignment between CuFeO_2_ and Cu_2_ZnSnS_4_ reduces energetic barriers to hole transport and limits interfacial recombination. Additionally, CuFeO2 contributes to visible-light absorption, thereby enhancing the overall PEC performance. The Cu_2_ZnSnS_4_ middle layer serves as both a light absorber and an electronic transport mediator. Importantly, the band positions of Cu_2_ZnSnS_4_ establish a graded energy cascade between CuFeO_2_ and Cs_2_SnCl_6_, facilitating upward electron flow and downward hole migration. This configuration reduces resistance to carrier transport across the multilayer stack. Cs_2_SnCl_6_, forming the top surface in direct contact with the electrolyte, operates as the terminal electron–delivery interface. It efficiently conducts, harvests photons, and accumulates electrons transferred from the underlying layers to drive the reduction of water-derived species.

The oxidation of water generates protons for hydrogen evolution and oxygen-containing intermediates.7
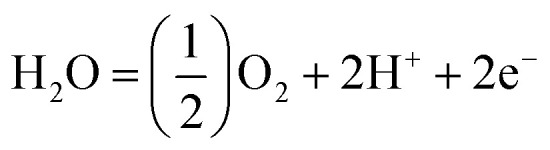


Electrons accumulated in the Cs_2_SnCl_6_ layer participate in proton reduction to generate H_2_ at catalytic sites.

8 2H^+^ + 2e^−^ = H_2_

Finally, the performance results of the current findings are compared with earlier research, as shown in Table 1, highlighting the advancements achieved by the CuFeO_2_/Cu_2_ZnSnS_4_/Cs_2_SnCl_6_ heterostructure.

**Table 1:** Comparison of PEC hydrogen generation parameters of the current study and the previously published works

Photoelectrode	*J*_ph_ mA cm^2^	Applied voltage (V)	Light source	Electrolyte	IPCE%
Au/PbS/Ro-GO/PANI^64^	1.1	+1	400 W xenon lamp	Na_2_S_2_O_3_	10.0
ZnO/TiO_2_/FeOOH^65^	1.59	0.8	A 150 W xenon lamp	Na_2_S_2_O_3_	—
TiO_2_/CdS/PbS^66^	2.0	0.2	AM 1.5G illumination	Na_2_S/Na_2_S_2_O_3_	4
CuO–C/TiO_2_ (ref. 67)	0.001	−0.5	300 W xenon lamp	Glycerol	—
g-C_3_N_4_/CuO^68^	0.01	1.6	300 W xenon lamp	NaOH	—
CsSnI_3_ (ref. 69)	0.19	0.01	Simulated AM1.5 illumination	*m*-MTDATA	3 × 10^−4^
*m*-CZTS^70^	23.15	1.127	Simulated AM1.5 sunlight	—	16.71
SnO_2_/TiO_2_ (ref. 71)	0.4	0.6	1 Sun (100 mW cm^−2^)	Na_2_S_2_O_3_	—
CuFeO_2_ (ref. 72)	1.25	0.9	1 sun illumination	1 M NaOH	—
CuFe_2_O_4_ (ref. 73)	0.04	1.1	1 sun illumination	0.1 M NaOH	—
CuFeO_2_/Cu_2_ZnSnS_4_/Cs_2_SnCl_6_ (this work)	24.0	1.0	Simulated sunlight	Sewage water	77.0 (at 390 nm)

To further enhance the performance of the proposed photoelectrode, our future studies will investigate the incorporation of plasmonic layers or two-dimensional (2D) materials within the multilayer heterostructure. Plasmonic nanostructures can amplify local electromagnetic fields, thereby improving light absorption and photon utilization. 2D materials, such as graphene or MXenes, provide exceptional electrical conductivity, a large surface area, and tunable band structures, which can promote faster charge transfer, minimize recombination losses, and enhance catalytic activity. These strategies are expected to further boost the PEC efficiency and long-term stability of the heterostructure, paving the way for more practical and scalable solar-driven hydrogen production systems.

## Conclusion

This study demonstrates the successful design and evaluation of a novel CuFeO_2_/Cu_2_ZnSnS_4_/Cs_2_SnCl_6_ heterostructure photoelectrode for efficient photoelectrochemical hydrogen production using real sewage water as the electrolyte. Systematic characterization confirmed enhanced crystallinity, strong light-harvesting capability, and a progressive reduction in bandgap energy. Under illumination, the heterostructure delivers photocurrent densities of up to 24.0 mA cm^−2^, compared with 0.31 mA cm^−2^ in the dark. A photon-to-current conversion efficiency (IPCE) of 77.0% was achieved, surpassing many previously reported systems. Thermodynamic analysis revealed favorable activation parameters for hydrogen evolution, with enthalpic (ΔH* = 3.452 kJ mol^−1^) and entropic (ΔS* = 9.644 J mol^−1^ K^−1^) values, underscoring the ability of the heterostructure to harness thermal energy in synergy with light absorption. Collectively, these findings establish the CuFeO_2_/Cu_2_ZnSnS_4_/Cs_2_SnCl_6_ photoelectrode as a promising candidate for sustainable solar-driven hydrogen production, even under complex wastewater conditions. Future work will focus on device-scale integration and long-term operational stability to advance practical applications in clean energy generation.

## Conflicts of interest

The authors confirm no conflict of interest.

## Funding statement

This work was supported and funded by the Deanship of Scientific Research at Imam Mohammad Ibn Saud Islamic University (IMSIU) (grant number IMSIU-DDRSP2503).

## Supplementary Material

NA-008-D5NA00828J-s001

## Data Availability

The data supporting this study's findings are available from the corresponding author upon reasonable request. Supplementary information is available. See DOI: https://doi.org/10.1039/d5na00828j.
