# Supplementary material for: All-lead-free Cs2SnCl6/Cu2ZnSnS4/CuFeO2 cascade band-aligned multilayer heterostructures for solar-driven hydrogen production from wastewater
Source: Nanoscale Adv. 2025 Nov 24;8(2):543–56. doi: 10.1039/d5na00828j (PMC12679471; doi:10.1039/d5na00828j)
Supplement: NA-008-D5NA00828J-s001 [file NA-008-D5NA00828J-s001.pdf]

Supplementary Information (SI) for Nanoscale Advances.
This journal is © The Royal Society of Chemistry 2025

All-lead-free $Cs_2SnCl_6/Cu_2ZnSnS_4/CuFeO_2$ cascade band-aligned multilayer heterostructures for solar-driven hydrogen production from wastewater

Amira H. Ali [1], Ashour M. Ahmed [2], Mohamed A. Basyooni-M. Kabatas [3,4*], Mamduh J. Aljaafreh [2], Mohamed Shaban [5], Mohamed Rabia [1], Ahmed A. Abdel-Khaliek [6]

[1] Nanomaterials Laboratory, Department of Chemistry, Faculty of Science, Beni-Suef University, Beni-Suef, Egypt

[2] Department of Physics, Faculty of Science, Imam Mohammad Ibn Saud Islamic University (IMSIU), Riyadh 11623, Saudi Arabia

[3] Department of Precision and Microsystems Engineering, Delft University of Technology, Mekelweg 2, 2628 CD Delft, The Netherlands

[4] Department of Nanotechnology and Advanced Materials, Graduate School of Applied and Natural Science, Selçuk University, Konya 42030, Turkey

[5] Department of Physics, Faculty of Science, Islamic University of Madinah, Madinah, Saudi Arabia

[6] Department of Chemistry, Faculty of Science, Beni-Suef University, Beni-Suef, Egypt

* Corresponding author: M.A. B.-M. K. (m.kabatas@tudelft.nl & m.a.basyooni@gmail.com).

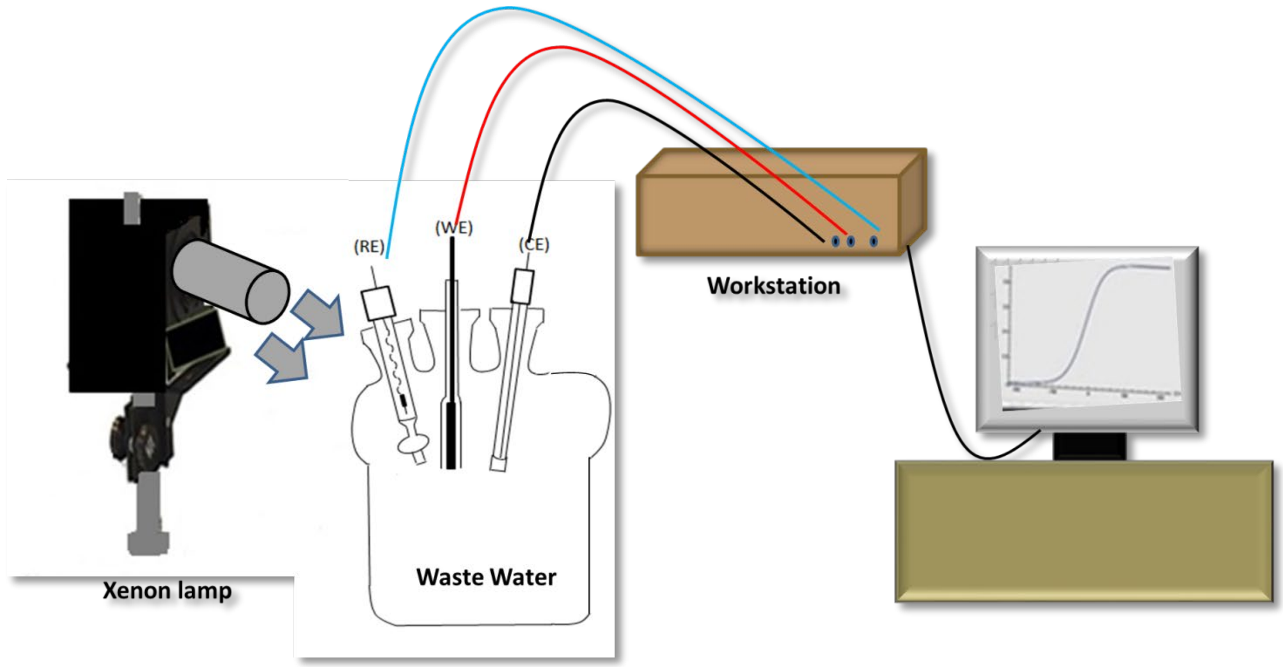

Figure S1. Schematic representation of the experimental setup used to evaluate the $CuFeO_2/Cu_2ZnSnS_4/Cs_2SnCl_6$-based hydrogen generation system.

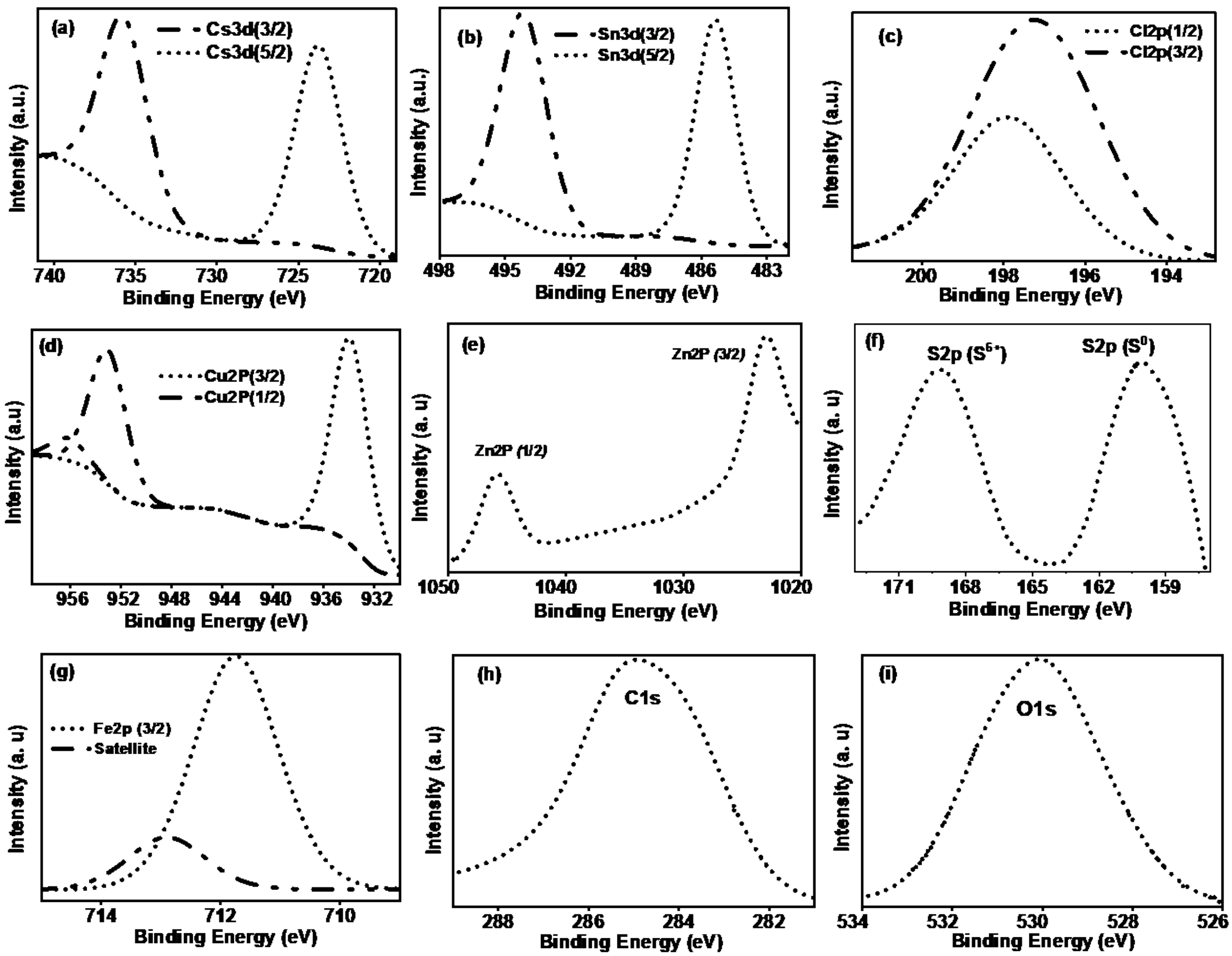

Figure S2. High-resolution XPS spectra of the $CuFeO_2/Cu_2ZnSnS_4/Cs_2SnCl_6$ multilayers.
